# Enhanced Thermoelectric Performance of *c*-Axis-Oriented Epitaxial Ba-Doped BiCuSeO Thin Films

**DOI:** 10.1186/s11671-018-2752-6

**Published:** 2018-11-28

**Authors:** Dachao Yuan, Shuang Guo, Shuaihang Hou, Yuejin Ma, Jianglong Wang, Shufang Wang

**Affiliations:** 1 Hebei Key Lab of Optic-electronic Information and Materials, The College of Physics Science and Technology, Hebei University, Baoding, 071002 China; 2 College of Mechanical and Electrical Engineering, Agricultural University of Hebei, Baoding, 071001 China

**Keywords:** BiCuSeO epitaxial thin films, Ba doping, Thermoelectric performance, Valence state of elements

## Abstract

We reported the epitaxial growth of *c*-axis-oriented Bi_1−*x*_Ba_*x*_CuSeO (0 ≤ *x* ≤ 10%) thin films and investigated the effect of Ba doping on the structure, valence state of elements, and thermoelectric properties of the films. X-ray photoelectron spectroscopy analysis reveal that Bi^3+^ is partially reduced to the lower valence state after Ba doping, while Cu and Se ions still exist as + 1 and − 2 valence state, respectively. As the Ba doping content increases, both resistivity and Seebeck coefficient decrease because of the increased hole carrier concentration. A large power factor, as high as 1.24 mWm^−1^ K^−2^ at 673 K, has been achieved in the 7.5% Ba-doped BiCuSeO thin film, which is 1.5 times higher than those reported for the corresponding bulk samples. Considering that the nanoscale-thick Ba-doped films should have a very low thermal conductivity, high *ZT* can be expected in the films.

**Electronic supplementary material:**

The online version of this article (10.1186/s11671-018-2752-6) contains supplementary material, which is available to authorized users.

## Background

The worldwide energy crisis and environmental issue in the past few decades triggered the need for renewable clean energy, and extensive efforts have been devoted to seek for innovative thermoelectric (TE) materials because of their potential applications in waste heat conversion and Peltier cooling. The performance of TE materials is qualified by the dimensionless figure of merit *ZT* = (*S*^*2*^/*ρκ*)*T*, where *S* is the Seebeck coefficient, *ρ* is the electrical resistivity, *κ* is the thermal conductivity, *T* is the absolute temperature. Therefore, to achieve a high *ZT*, one strategy is to improve the power factor *S*^*2*^/*ρ* and another is to suppress the thermal conductivity *κ*.

BiCuSeO, a quaternary oxyselenide, has attracted great attention as a new promising TE material due to its intrinsically very low thermal conductivity [1, 2]. This compound crystallizes in a tetragonal ZrCuSiAs structure with P4/nmm space group, which consists of the insulating (Bi_2_O_2_)^2+^ layers and the conductive (Cu_2_Se_2_)^2−^ layers alternatively stacked along the *c* axis. Over the past several years, extensive works have been done in enhancing the TE performance of BiCuSeO bulks by optimization of its power factor and thermal conductivity via element doping [3–13], *c*-axis texturing [14], band gap tuning [15, 16], creating Bi or/and Cu vacancies [17–19], engineering grain boundaries [20, 21], adding nano-inclusions [22], introducing spin entropy by magnetic ion [23], and etc. For example, Zhao LD et al. reported a high *ZT* of about 1.4 at 923 K in the *c*-axis-textured Ba-doped BiCuSeO bulks. The texturation significantly optimized the carrier mobility, leading to the increase in electrical conductivity and thus the power factor [14]. Xie X et al. explored the high performance of BiCuSeO bulks via introducing Bi/Cu dual vacancies, and a high *ZT* value of 0.84 was obtained at 750 K. Dual vacancies greatly suppressed the thermal conductivity owing to the increased phonon scattering. Moreover, the interlayer charge transfer between these Bi/Cu dual vacancies resulted in the significant increase of electrical conductivity with relatively high Seebeck coeffecient [19]. Recently, Liu. Y et al. reported the synergistically optimizing electrical and thermal transport properties of BiCuSeO bulks via a Pb/Ca dual-doping approach, and a record high *ZT* of about 1.5 was achieved in the sample of Bi_0.88_Ca_0.06_Pb_0.06_CuSe at 873 K [12].

For miniaturizing the TE devices, nanoscale thin film may be advantageous because it is compatible with microelectromechanical system. Moreover, thin film TE devices can achieve very high cooling power densities and very fast cooling [24, 25]. However, BiCuSeO-based thin film fabrication is challenging due to the difficulty of controlling a stoichiometry transfer of such complex structures and the presence of volatile elements of Bi and Se. So far, there have been only very few reports on thin film growth and thermoelectric properties [26–28]. In this paper, *c*-axis-oriented Ba-doped BiCuSeO thin films were grown on SrTiO_3_ (001) substrates by pulsed laser deposition, and the effects of Ba doping on the structure, valence state of elements, and TE properties of the films were investigated. On the one hand, Ba^2+^ can effectively substitute Bi^3+^ as a *p*-type dopant, resulting in the optimized electrical transport properties of BiCuSeO owing to the increase of the carrier concentration. On the other hand, Ba doping can introduce Ba-Bi substitutional point defects, which can effectively scatter high-frequency phonons and greatly reduce the lattice thermal conductivity of BiCuSeO. A best power factor (PF) 1.24 mW m^−1^ K^−2^ at about 673 K has been achieved in the 7.5% Ba-doped thin film sample, which is about 1.5 times higher than those reported for the Pb/Ca dual-doped or *c*-axis-textured Ba-doped BiCuSeO bulk samples. Since nanoscale-thick thin films usually have very low thermal conductivity, high thermoelectric performance can be expected in these Ba-doped BiCuSeO thin films.

## Methods

The Bi_1 − *x*_Ba_*x*_CuSeO (*x* = 0%, 2.5%, 5%, 7.5%, 10%) thin films with thickness of about 50 nm were deposited on the commercial SrTiO_3_ (001) single crystalline substrates by PLD technique under high purity argon atmosphere. The in-plane lattice parameters of BiCuSeO (*a* = *b* = 0.3926 nm) are very close to those of SrTiO_3_ (cubic, *a* = *b* = 0.3905 nm), leading to a small in-plane lattice mismatch of about 0.54% between the film and substrate. An excimer laser with the wavelength of 308 nm was used for ablation of the corresponding polycrystalline ceramic targets sintered by the traditional solid-state reaction method in vacuum-sealed quartz tubes. During the film growth, the laser energy density on the target was about 1.0 J cm^−2^, the repetition rate of the laser was 5 Hz, the distance between the film and the substrate was about 50 mm, the argon pressure was about 0.1 Pa, and the substrate temperatures was about 330 °C, respectively.

The crystal structure of the film was measured using X-ray diffraction (XRD) with Cu K_α_ radiation. The surface morphology was analyzed by scanning electron microscope (SEM, FEI XL30 S-FEG) with an operating voltage of 15 kV. A field-emission transmission electron microscope (TEM, Tecnai G2 F20) was used to characterize the microstructural properties of the films. X-ray photoelectron spectroscopy (XPS, PHI Quantera SXM, ULVAC-PHI, Japan) was used to investigate the valence states of the elements. The XPS measurements were performed ex situ. The working pressure in the XPS chamber was approximately 2 × 10^−7^ Pa. Before the measurement, the sample was etched for about 5 min by low energy Ar^+^ in the XPS chamber to remove the impurities on the film surface. Hall measurements were performed in a physical property measurement system (PPMS-9) by using the van der Pauw configuration. The four-probe electrical resistivity and the Seebeck coefficient were measured in helium using a commercial equipment (Linseis, LSR-800) from room temperature to 700 K with the heating rate of 5 K min^−1^.

## Results and Discussion

**Fig. 1:**
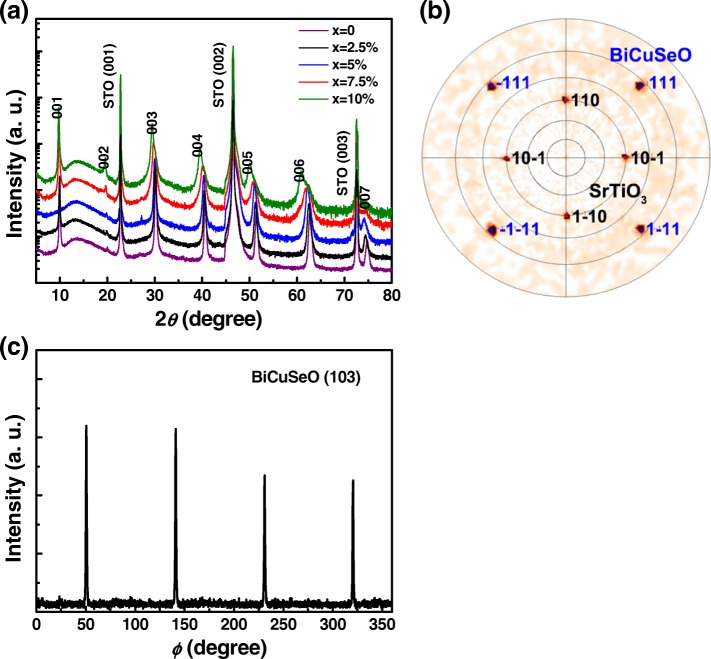
**a** XRD *θ*–2*θ* scans of Bi_1 − *x*_Ba_*x*_CuSeO (0 ≤ *x* ≤ 10%) thin films on the SrTiO_3_(001) substrates. **b** Pole figure of BiCuSeO (111) and SrTiO_3_ (110) recorded at 33.75°. **c** φ scan of the (103) peak of BiCuSeO thin film sample

Figure 1a shows the XRD *θ*–2*θ* scans of Bi_1 − *x*_Ba_*x*_CuSeO thin films with different Ba-doping content. All peaks in the patterns can be indexed to (00 *l*) diffractions of tetragonal BiCuSeO phase with space group P4/nmm (PDF #45–0296), indicating that BiCuSeO films with perfect *c*-axis alignment are obtained. The full width at half maxima of these diffraction peaks increases with the increase of Ba-doping content, revealing that the average grain size in the films becomes smaller. The reduction of grain size is most likely due to the pinning effect of dopant which can suppress the movement of grain boundaries of BiCuSeO and thus inhibit the grain growth [29, 30]. In addition, an obvious shift of 2*θ* towards the smaller angle is observed with the increase of Ba-doping content owing to the larger ionic radii of Ba^2+^(1.42 Å) in comparison with Bi^3+^(1.17 Å), which suggests that Ba^2+^ was successfully incorporated into the BiCuSeO lattice at the Bi^3+^ site. The *c* lattice parameters of the present BiCuSeO thin films calculated from XRD results of Fig. 1a show an increase trend with the Ba content, and the values are very close to the corresponding bulk samples [8]. Recently, He et al. investigated the Ba heavily doped BiCuSeO bulk samples with Ba-doping content ≥ 5% via Cs-corrected STEM, and found only part of Ba dopant substituted Bi atoms in Bi–O layers and exceeding Ba formed some nanoscale BaSeO_3_ precipitates dispersed in the BiCuSeO matrix [6]. However, no obvious second phases are detected in the present Ba heavily doped BiCuSeO thin films within the XRD measurement limit, which might be due to the solubility limit of Ba is higher in the PLD-fabricated films.

The *ab* plane texture information was investigated by XRD pole figures using a Bruker D8 diffractometer with the GADDS system. We recorded one at 2*θ* = 33.75°. This particular value of the angle was chosen because (i) it corresponds to a high-intensity peak of the BiCuSeO structure, namely, the (111) peak, (ii) it is close to the (110) peak of the SrTiO_3_ substrate, enabling to observe both components of BiCuSeO and SrTiO_3_ on the same pole figure. The result is given in Fig. 1b for both observed and simulated ones. The analysis was performed by visual comparison of the measured enhanced pole densities with calculated spherical projections of SrTiO_3_ and BiCuSeO crystals, using the software STEREOPOLE [31]. Firstly, the different poles observed can be simulated considering an (00 *l*) oriented BiCuSeO film deposited on an (100) SrTiO_3_ substrate (as already inferred from *θ*–2*θ* scans); secondly, since only punctual poles are observed, one can conclude that the film is not only textured but epitaxial. Finally, the different orientations between the two simulated lattices lead to the following epitaxial relationships between the SrTiO_3_ substrate and the BiCuSeO film: [010] SrTiO_3_//[010] BiCuSeO and [001] SrTiO_3_//[− 100] BiCuSeO. We also performed the phi scan measurements for the film, as shown in Fig. 1c. It can be clearly seen that the phi scan exhibits 4-fold symmetric diffraction peaks, corresponding to the tetragonal symmetry of the lattice.

**Fig. 2:**
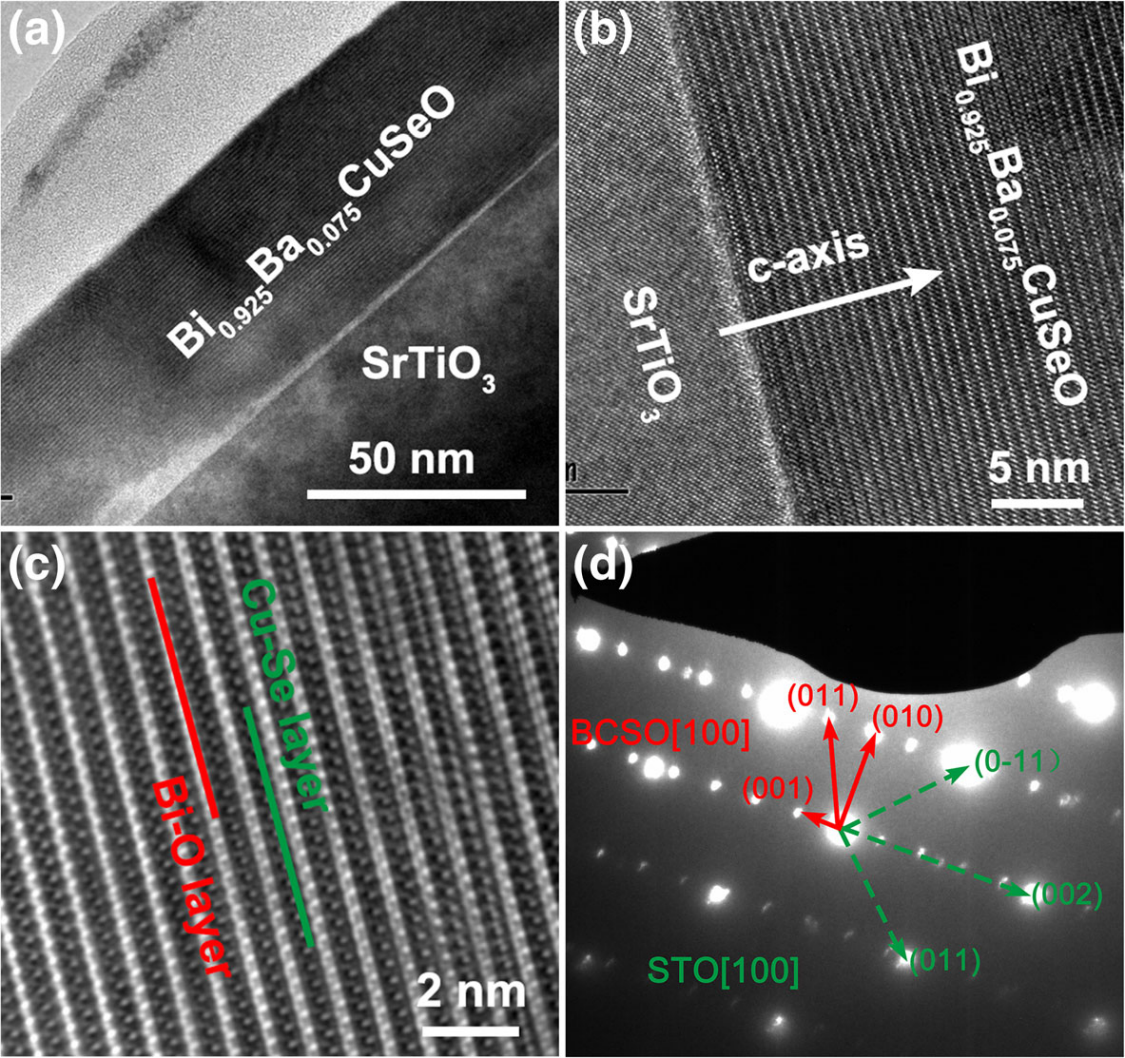
**a** Low- and (**b**) high-magnification cross-sectional TEM image of a Bi_0.925_Ba_0.075_CuSeO film on SrTiO_3_ (001) substrate. **c** The magnified HRTEM image of the film part. **d** The corresponding SEAD pattern of the Bi_0.925_Ba_0.075_CuSeO/SrTiO_3_ cross section. The electron beam incident direction in **a**–**d** is all along the [001] direction

Figure 2a displays the cross-sectional low-resolution TEM images of a 7.5% Ba-doped BiCuSeO thin film samples on SrTiO_3_ substrate, exhibiting very flat surface and interface. A very thin “bright” layer with the thickness of a few nanometer can be observed at the interface between film and substrate, which could be induced by the crystallographic structure mismatch of the two heterogeneous phases since the film growth temperature is relatively low [32]. Figure 2b, c shows the cross-sectional high-resolution TEM image of the same sample. A layered structure with alternately stacked Bi-O insulating layers and Cu-Se conducting layers along the *c*-axis is clearly visible in the images. Figure 2d shows the corresponding selected area electron diffraction (SAED) pattern, which confirms the *c*-axis-oriented epitaxial nature of the film on SrTiO_3_ substrate.

**Fig. 3:**
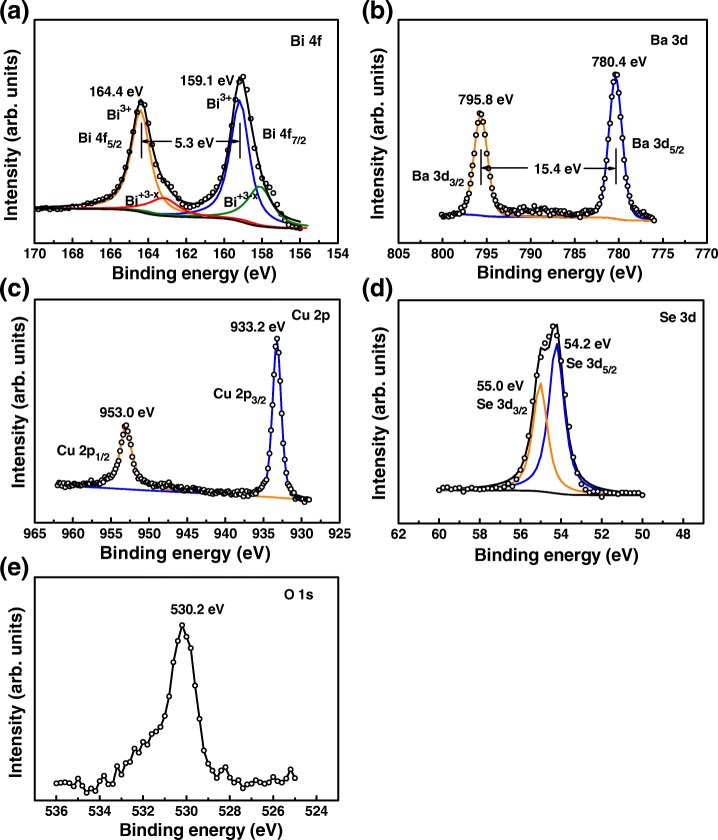
XPS spectra of **a** Bi 4f, **b** Ba 3d, **c** Cu 2p, **d** Se 3d, and **e** O 1s in Bi_0.925_Ba_0.075_CuSeO thin film

The valence states of the ions in BiCuSeO film after Ba doping were analyzed by XPS. Figure 3a–d presents the XPS core level spectra of Bi 4f, Ba 3d, Cu 2p, and Se 3d of the 7.5% Ba-doped BiCuSeO thin film sample, respectively. The C 1 s (284.8 eV) line was used to calibrate the binding-energy scale for XPS measurements. Figure 3a exhibits two main peaks at the binding energy of 159.1 and 164.4 eV, corresponding to the core lines of 4f_7/2_ and 4f_5/2_ of Bi^3+^ ions, respectively. The binding energy difference between these two peaks is about 5.3 eV, which is in good agreement with the previously data obtained from the Pb or Ca-doped BiCuSeO bulk samples [10, 33]. Moreover, additional shoulder peaks located at the lower binding energy side of the Bi^3+^ peak are observed in Fig. 3a, indicating that there exist some Bi ions with lower oxidation state of + 3 − *x* in the Ba-doped film sample [10, 33]. These Bi ions with lower valence state can contribute holes to Cu-Se layer, thus increasing the carrier concentration and improving the electrical conductivity. The core level spectrum of Ba 3d reveals that Ba tends to oxidize to a stable + 2 oxidation state in the Bi_0.925_Ba_0.075_CuSeO film. As shown in Fig. 3b, the peaks at binding energy 780.4 and 795.8 eV can be assigned to Ba3d_5/2_ and 3d_3/2_ core lines of Ba^2+^, respectively [34]. Figure 3c presents the Cu 2p core level spectrum of Bi_0.925_Ba_0.075_CuSeO thin film. It can be observed that the Cu 2p_3/2_ and Cu 2p_1/2_ peaks locate at 933.2 eV and 953.0 eV respectively, with a significant binding energy difference of about 19.8 eV. The peaks are symmetric, and there is no visible satellite. This result suggests that the Cu ion exists mainly as Cu^+^ in the present Ba-doped thin films [35]. The Se 3d core level spectrum in Fig. 3d can be fitted with two peaks at binding energy of 54.2 and 55.0 eV, corresponding to the Se 3d_5/2_ and 3d_3/2_ of Se^2−^, respectively [36, 37]. Figure 3e shows the O 1s core level spectrum of the film. It exhibits a peak at the binding energy of about 530.2 eV, corresponding to the oxygen chemical state of − 2. The single O 1s peak with a small high-binding energy shoulder reflects the cleanliness of the sample surface [38]. Based on the XPS results, there should exist more hole carriers in the heavily doped films, which can be confirmed later.

**Fig. 4:**
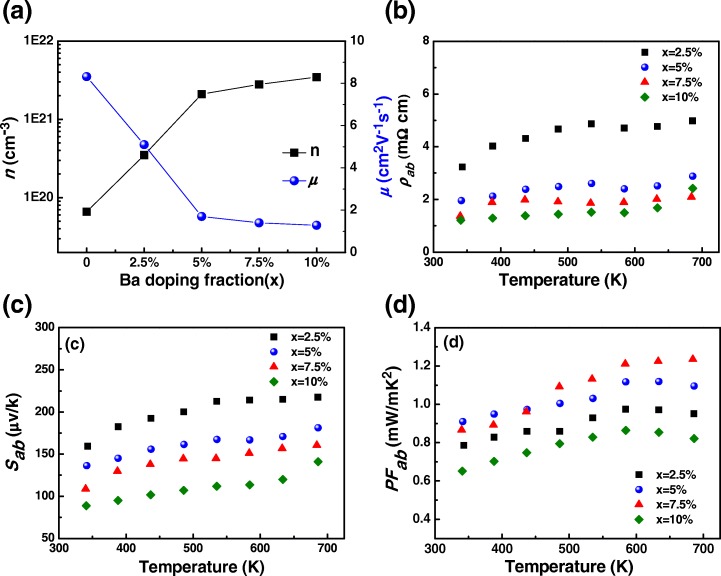
**a** Carrier concentration *n* and mobility *μ* of the Bi_1 − *x*_Ba_*x*_CuSeO (0 ≤ *x* ≤ 10%) thin films measured at room temperature. **b** The temperature dependence of the *ab* plane resistivity *ρ*_*ab*_. **c** Seebeck coefficient *S*_*ab*_. **d** Power factor PF_*ab*_ of the Bi_1 − *x*_Ba_*x*_CuSeO (0 ≤ *x* ≤ 10%) thin films

Hall measurements reveal that the major carriers in those films are holes. Figure 4a shows the variation of the room temperature carrier concentration *n* and mobility *μ* of the *c*-axis-oriented epitaxial Bi_1 − *x*_Ba_*x*_CuSeO films on the Ba-doping content. The good linearity of Hall voltage vs. external magnetic field can be found in the Additional file 1: Figure S1. The undoped film has the room temperature carrier concentration *n* of about 6.6 × 10^19^ cm^−3^, which is nearly an order of magnitude larger than those previously reported in the most bulk samples [5]. The higher *n* may originate from Cu or Bi vacancies in the films which can contribute holes [17–19]. As the Ba-doping content increases, the hole carrier concentration *n* of the films increases owing to the substitution of Bi^3+^ by Ba^2+^. Assuming that each Ba atom introduces one hole to BiCuSeO, the hole carrier concentration of the 2.5, 5, 7.5, and 10% Ba-doped films is calculated to be 3.62 × 10^20^, 7.25 × 10^20^, 1.08 × 10^21^, and 1.45 × 10^21^ cm^−3^, respectively. For samples with higher doping content (≥ 5%), the measured hole carrier concentration *n* is slightly larger than that of the calculated one, suggesting that there exist more Cu or Bi vacancies in the heavily doped films. As for the carrier mobility, it decreases from 8.3 cm^2^ V^−1^ s^−1^ for the undoped film to 1.3 cm^2^ V^−1^ s^−1^ for the 10% Ba-doped film because of the enhanced carrier scatterings. It should be noted that the Hall mobility *μ* obtained in the present BiCuSeO thin films is relatively high irrespective of heavy doping. Similar large Hall mobility has also been obtained by Hidenori et al. in the epitaxial thin film of Mg-doped LaCuSeO, a compound with the same degenerate state as that of BiCuSeO, and can be attributed to the enhancement of chemical bond-covalency and hybridization of the relevant anion orbitals [39, 40]. Moreover, the band dispersion close to the VBM is larger in BiCuSeO than in LaCuSeO [41], which will lead to smaller effective mass and larger Hall mobility.

Figure 4b, c presents the *ab* plane electrical resistivity *ρ*_*ab*_ and Seebeck coefficient *S*_*ab*_ of the *c*-axis-oriented epitaxial Ba-doped BiCuSeO thin films measured above the room temperature, and the clear linearity of Δ *T* vs. Δ *V* in Seebeck coefficient measurements can be seen in the Additional file 1: Figure S2. The corresponding data of the undoped BiCuSeO thin film have not been provided here due to the high temperature resistance of this sample is beyond the maximum measurement limit of the LSR-800 system. However, we measured the room temperature *ρ*_*ab*_ and *S*_*ab*_ of the undoped BiCuSeO thin film by PPMS, which is about 12.5 mΩ cm and 201 μV K^−1^, respectively. The positive *S* values, as shown in Fig. 4c, reveal that the films are *p*-type conducting which are consistent with the Hall measurements. Figure 4b, c shows both *ρ*_*ab*_ and *S*_*ab*_ of each Ba-doped BiCuSeO thin film exhibit an increase trend with the increase of temperature, indicating a metallic-like conducting behavior. As the Ba-doping content *x* increases, both *ρ*_*ab*_ and *S*_*ab*_ of the Bi_1 − *x*_Ba_*x*_CuSeO film decrease owing to the increased hole carrier concentration. Moreover, due to the highly *c*-axis-orientated feature, the resistivity of all films is much smaller than that of the corresponding polycrystalline ceramics [3–19, 22, 23]. This can be explained by the anisotropy of the BiCuSeO system with a layered crystal structure, in which the resistivity in the *ab* plane is much lower than that along the *c*-axis direction [14].

Combining the electrical resistivity and Seebeck coefficient, the resultant power factor PF_*ab*_ (PF_*ab*_ = *S*_*ab*_^2^/*ρ*_*ab*_) of all film samples has been significantly improved as compared to those reported for the Ba-doped polycrystalline ceramics in the literatures [5, 11]. The maximum power factor of about 1.24 mW m^−1^ K^−2^ at 673 K has been obtained in the film sample of Bi_0.925_Ba_0.075_CuSeO (*ρ*_*ab*_ *~* 2.08 mΩ cm and *S*_*ab*_ ~ 161 μV K^−1^ for this sample at 673 K), as shown in Fig. 4d, which is nearly 2.8 times larger than that of the undoped film sample and about 1.5 times higher than the best results reported for the Pb/Ca dual-doped or *c*-axis-textured Ba-doped BiCuSeO bulk samples. The high power factor is mainly owing to the low resistivity of the film, which is induced by its high carrier concentration as well as the *c*-axis-oriented nature of the film. We also estimated *ZT* of the present BiCuSeO thin films. Here, the *ab* plane carrier thermal conductivity *κ*_e(*ab*)_ of the films was calculated from our experimental data according to the Wiedemann-Franz law (*κ*_*e*_ *= LT/ρ, L* is the Lorenz number), and the *ab* plane phonon thermal conductivity *κ*_*ph*(*ab*)_ was cited from the value reported in the corresponding *c*-axis-textured bulks (~ 0.55 and 0.35 W m^−1^ K^−1^at 300 K and 673 K, respectively, Energy *Environ. Sci.*, 2013, 6, 2916). The estimated *ZT* is about 0.26 at 300 K for the 7.5% Ba-doped film, and it reaches to 0.93 at the highest record temperature of 673 K. In fact, the *ZT* values of the present BiCuSeO films could be underestimated since the phonon thermal conductivity of the films is normally much lower than that of the corresponding bulk samples due to the strong phonon scatterings at the film surface as well as at the film/substrate interface, especially for a film with the thickness in the order of a few tens of nanometer [42, 43]. It should be mentioned here that the TE transport properties of thin films strongly depend on the film thickness. For semiconductor TE thin films, decreasing thickness normally results in an increase in resistivity and a decrease in Seebeck coefficient as well as the thermal conductivity. Detailed investigation of the thickness-dependent TE performance of the BiCuSeO thin films will be carried out in our coming work.

**Fig. 5:**
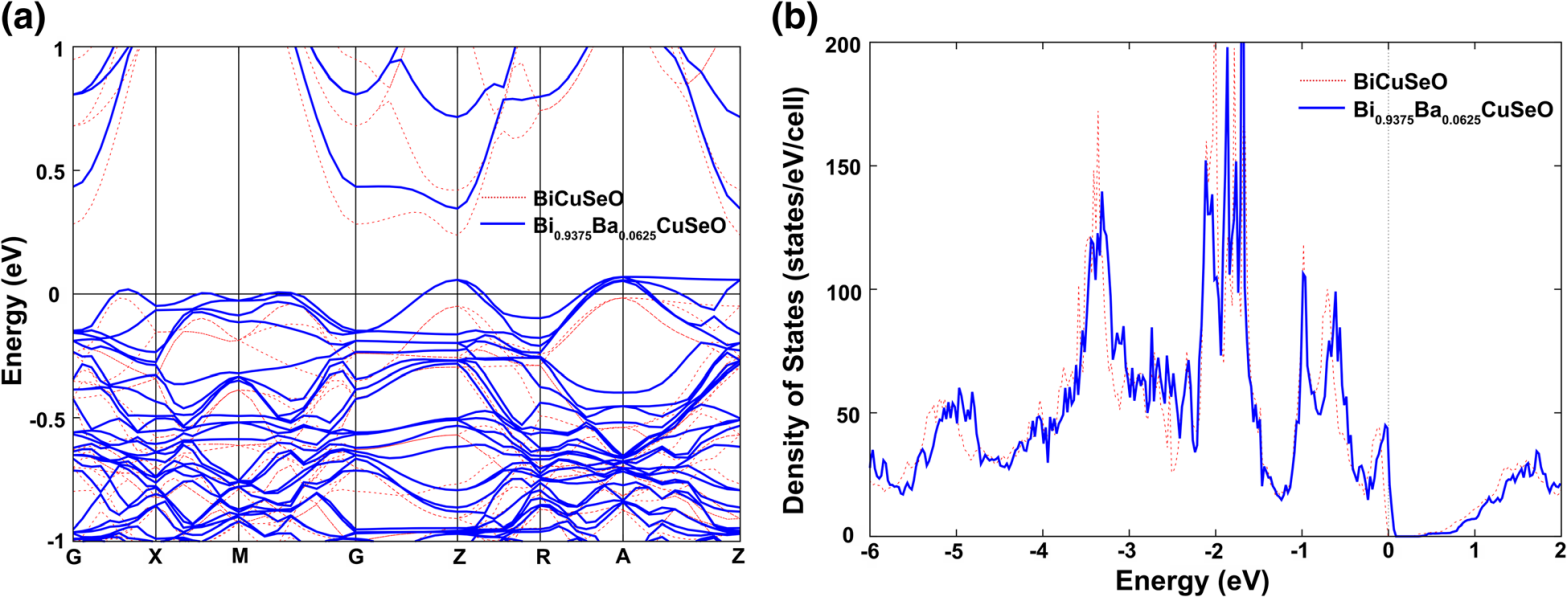
**a** Band structure. **b** Density of states of the pristine and Ba-doped BiCuSeO

The better understand the effect of Ba doping on the thermoelectric properties of BiCuSeO, we also calculated the band structure and the density of states of the pristine and Ba-doped BiCuSeO. The calculations were done by using the projector augmented wave (PAW) method as implemented in Vienna ab initio Simulation Package (VASP). The Perdew-Burke-Ernzerh generalized gradient approximation (PBE) to exchange–correlation potential was used for the optimization of the lattice constant and internal coordinates of the pristine BiCuSeO and the 64-atom supercell with one Bi atom substituted by one Ba atom (i.e., Bi_0.9375_Ba_0.0625_CuSeO). Figure 5a shows the band structures of the 64-atom supercell with and without one Ba atom substitution (only bands near the Fermi level are shown), their band structure exhibits almost the same dispersion, except the band degeneracy at some high symmetry point in the Brilliouin zone is lifted due to the reduced symmetry in the doped cell. With Ba substitution, the Fermi level moves into the valence bands consisting of Cu-3d and Se-4p orbital, suggesting the hole is introduced to the Cu-Se layers. The density of states of two supercells (Fig. 5b) also shows the similar shape and peak position, indicating rigid band-like behavior produced by Ba doping. The calculation results indicate that the valence band of BiCuSeO is less affected by Ba doping, and the enhanced power factor in Bi_1−*x*_Ba_*x*_CuSeO samples is mainly attributed to the increased hole carrier concentration induced by Ba doping.

## Conclusions

Bi_1 − *x*_Ba_*x*_CuSeO (0 ≤ *x* ≤ 10%) thin films were grown on SrTiO_3_ (001) substrates by PLD technique, and the effect of Ba doping on the thermoelectric properties of the films were investigated. X-ray diffraction and transmission electron microscope analysis revealed that the resulting films were *c*-axis-oriented with the in-plane epitaxial relationships between the film and the substrate of [010] BiCuSeO//[010] SrTiO_3_ and [− 100] BiCuSeO//[001] SrTiO_3_. With the increase of Ba-doping content from 0 to 10%, both the resistivity and Seebeck coefficient of the films decreased, primarily owing to the increased hole carrier concentration induced by the substitution of Ba^2+^ for Bi^3+^. Benefiting from the low resistivity, all films exhibit larger power factors than those previously reported in the corresponding polycrystalline bulk samples. The highest power factor of 1.24 mW m^−1^ K^−2^ at 673 K has been obtained in the 7.5% Ba-doped thin film sample, which is nearly 2.8 times larger than that of the undoped film sample and 1.5 times higher than the corresponding Ba-doped bulk samples. Considering the fact that the nanoscale-thick films have a very low thermal conductivity as well as Ba doping can further suppress the lattice thermal conductivity, high thermoelectric performance can be expected in the present Ba-doped BiCuSeO thin films.

## Additional File

Additional file 1: **Figure S1.** The linear dependency of Hall voltage on the external magnetic field. **Figure S2.** The voltage difference (Δ*V*) vs. temperature difference (Δ*T*) plot for the Seebeck coefficient measured at 340 K of the Bi_0.975_Ba_0.025_CuSeO thin film. (DOCX 90 kb)

## Abbreviations

PLD: Pulsed laser deposition
PPMS: Physical property measurement system
SAED: Selected area electron diffraction
SEM: Scanning electron microscope
TE: Thermoelectric
TEM: Transmission electron microscope
XPS: X-ray photoelectron spectroscopy
XRD: X-ray diffraction
