# Supplementary material for: Enhanced Thermoelectric Performance of c-Axis-Oriented Epitaxial Ba-Doped BiCuSeO Thin Films
Source: Nanoscale Res Lett. 2018 Nov 28;13:382. doi: 10.1186/s11671-018-2752-6 (PMC6261905; doi:10.1186/s11671-018-2752-6)
Supplement: Supplementary file 1 — Figure S1. The linear dependency of Hall voltage on the external magnetic field. Figure S2. The voltage difference (ΔV) vs. temperature difference (ΔT) plot for the Seebeck coefficient measured at 340 K of the Bi0.975Ba0.025CuSeO thin film. (DOCX 90 kb) [file 11671_2018_2752_MOESM1_ESM.docx]

Enhanced thermoelectric performance of *c*-axis oriented epitaxial Ba-doped BiCuSeO thin films

(SUPPLEMENTARY MATERIAL)

Dachao Yuan^1,2^, Shuang Guo^1^*, Shuaihang Hou^1^, Yuejin Ma^2^, JiangLong Wang^1^ and Shufang Wang^1^*

^1^Hebei Key Lab of Optic-electronic Information and Materials, The College of Physics Science and Technology, Hebei University, Baoding 071002, China.

^2^College of Mechanical and Electrical Engineering, Agricultural University of Hebei, Baoding 071001, China.

*Corresponding authors E-mail: [gshuang123@126.com](mailto:gshuang123@126.com) (Shuang Guo), [sfwang@hbu.edu.cn](mailto:sfwang@hbu.edu.cn) (Shufang Wang)

**Figure S1.** The linear dependency of Hall voltage on the external magnetic field.

**Figure S2**. The voltage difference (ΔV) vs. temperature difference (ΔT) plot for the Seebeck coefficient measured at 340 K of the Bi_0.975_Ba_0.025_CuSeO thin film.
